# Phenotyping of multiple sclerosis lesions according to innate immune cell activation using 18 kDa translocator protein-PET

**DOI:** 10.1093/braincomms/fcab301

**Published:** 2021-12-22

**Authors:** Marjo Nylund, Marcus Sucksdorff, Markus Matilainen, Eero Polvinen, Jouni Tuisku, Laura Airas

**Affiliations:** Turku PET Centre, Turku, Finland; Clinical Neurosciences, University of Turku, Turku, Finland; Neurocenter, Turku University Hospital, Turku, Finland; Turku PET Centre, Turku, Finland; Clinical Neurosciences, University of Turku, Turku, Finland; Neurocenter, Turku University Hospital, Turku, Finland; Turku PET Centre, Turku, Finland; Faculty of Science and Engineering, Åbo Akademi University, Turku, Finland; Turku PET Centre, Turku, Finland; Clinical Neurosciences, University of Turku, Turku, Finland; Neurocenter, Turku University Hospital, Turku, Finland; Turku PET Centre, Turku, Finland; Turku PET Centre, Turku, Finland; Clinical Neurosciences, University of Turku, Turku, Finland; Neurocenter, Turku University Hospital, Turku, Finland

**Keywords:** PET imaging, TSPO, multiple sclerosis, innate immune cell, white matter lesions

## Abstract

Chronic active lesions are promotors of neurodegeneration and disease progression in multiple sclerosis. They harbour a dense rim of activated innate immune cells at the lesion edge, which promotes lesion growth and thereby induces damage. Conventional MRI is of limited help in identifying the chronic active lesions, so alternative imaging modalities are needed. Objectives were to develop a PET-based automated analysis method for phenotyping of chronic lesions based on lesion-associated innate immune cell activation and to comprehensively evaluate the prevalence of these lesions in the various clinical subtypes of multiple sclerosis, and their association with disability. In this work, we use 18 kDa translocator protein-PET imaging for phenotyping chronic multiple sclerosis lesions at a large scale. For this, we identified 1510 white matter T1-hypointense lesions from 91 multiple sclerosis patients (67 relapsing–remitting patients and 24 secondary progressive patients). Innate immune cell activation at the lesion rim was measured using PET imaging and the 18 kDa translocator protein-binding radioligand ^11^C-PK11195. A T1-hypointense lesion was classified as rim-active if the distribution volume ratio of ^11^C-PK11195-binding was low in the plaque core and considerably higher at the plaque edge. If no significant ligand binding was observed, the lesion was classified as inactive. Plaques that had considerable ligand binding both in the core and at the rim were classified as overall-active. Conventional MRI and disability assessment using the Expanded Disability Status Scale were performed at the time of PET imaging. In the secondary progressive cohort, an average of 19% (median, interquartile range: 11–26) of T1 lesions were rim-active in each individual patient, compared to 10% (interquartile range: 0–20) among relapsing–remitting patients (*P* = 0.009). Secondary progressive patients had a median of 3 (range: 0–11) rim-active lesions, versus 1 (range: 0–18) among relapsing–remitting patients (*P* = 0.029). Among those patients who had rim-active lesions (*n* = 63), the average number of active voxels at the rim was higher among secondary progressive compared to relapsing–remitting patients (median 158 versus 74; *P* = 0.022). The number of active voxels at the rim correlated significantly with the Expanded Disability Status Scale (*R* = 0.43, *P* < 0.001), and the volume of the rim-active lesions similarly correlated with the Expanded Disability Status Scale (*R* = 0.45, *P* < 0.001). Our study is the first to report *in vivo* phenotyping of chronic lesions at large scale, based on 18 kDa translocator protein-PET. Patients with higher disability displayed a higher proportion of rim-active lesions. The *in vivo* lesion phenotyping methodology offers a new tool for individual assessment of smouldering (rim-active) lesion burden.

## Teaser

This study describes *in vivo* phenotyping of multiple sclerosis lesions using translocator protein-PET. Nylund *et al*. report that secondary progressive multiple sclerosis patients displayed high proportion of rim-active lesions, indicating greater innate immune cell activation at the chronic lesion edge. The methodology offers a new tool for individual assessment of smouldering multiple sclerosis lesion burden.

## Graphical Abstract

**Graphical Abstract:**
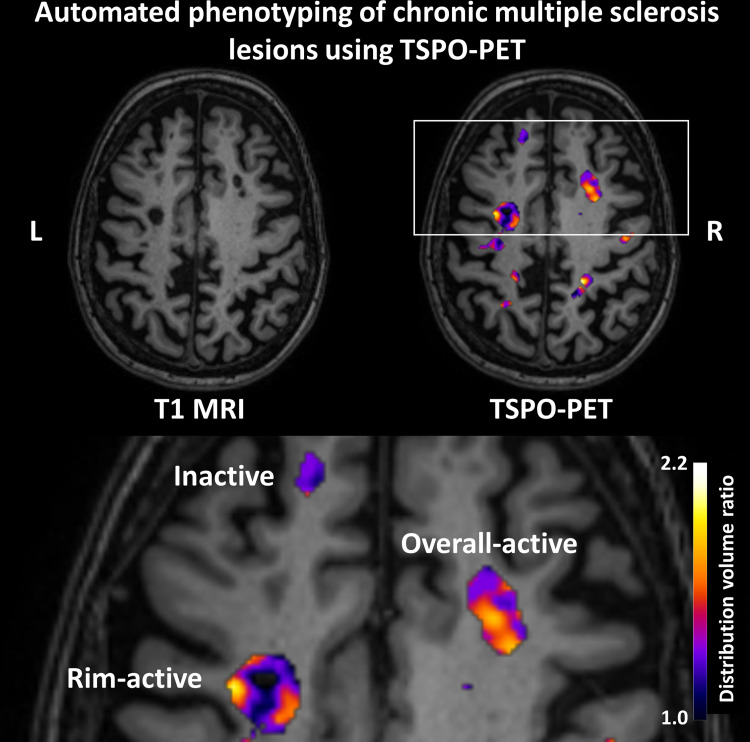

## Introduction

Despite appropriate use of disease-modifying therapies, the majority of relapsing–remitting multiple sclerosis (RRMS) patients proceed to secondary progressive disease course characterized by gradual accumulation of disability and paucity of relapses.^1^ In secondary progressive multiple sclerosis (SPMS), immune cell trafficking from the periphery is reduced and neuropathological studies reveal chronic and compartmentalized activation of the innate immune system within the CNS behind an intact blood–brain barrier and fewer active focal lesions.^2^ In clinical imaging, this translates to fewer MRI-detectable acute gadolinium-enhancing plaques and more abundant chronic T1-hypointense lesions. In SPMS, the predominant lesion type in neuropathological studies is a chronic lesion. These typically have an acellular lesion core, and lesion edge not containing (inactive) or containing activated microglial cells and macrophages (chronic active, i.e. smouldering plaques).^3,4^ The smouldering lesions are frequently associated with signs of axonal damage and demyelination.^5^ The smouldering lesion-associated innate immune cells have a proinflammatory phenotype and increased iron uptake,^6^ which makes them visible *in vivo* by MRI sequences sensitive to tissue susceptibility.^7^ Clinical imaging studies have recently identified the iron rim lesions as a marker of poor prognosis, with associated lesion growth and more rapid clinical disease progression.^8–10^ Depending on the study, the iron rim lesion fraction has varied between 0 and 75% per patient.^8,9,11–13^

PET imaging using radioligands binding to the 18 kDa translocator protein (TSPO) has similarly been used to specifically quantitate innate immune cell activation *in vivo*, and increased PET-detectable TSPO-expression in lesions with paramagnetic rims has been demonstrated.^13^ TSPO-PET signal is stronger both in the normal appearing white matter (NAWM) and in the perilesional area of SPMS patients compared to RRMS patients and controls^14–19^ and upon longitudinal follow-up, an increase in TSPO-binding in the NAWM was observed in an untreated group of multiple sclerosis patients studied using PET.^20^ In lesional areas of multiple sclerosis brain, gadolinium-enhancing lesions have strong TSPO–ligand accumulation,^21^ whereas non-enhancing T2 lesions have more variable TSPO-binding patterns both within the lesion and in the perilesional area.^16,17^ There is yet no comprehensive *in vivo* PET-based analysis of innate immune cell activity at the chronic lesion rim. It would be advantageous to be able to define the frequency of smouldering lesions at different stages of the disease, to better understand their impact on disease progression, to shed light to the risk of progression and disability accrual and to be able to measure more accurately the impact the innate immune system-modifying therapies within the CNS *in vivo*.

The aim of this study was to develop an automated TSPO-PET analysis method for comprehensive phenotyping of individual chronic lesions based on their microglial activation status *in vivo*. Using this method, we determined the proportions of different lesion types in various multiple sclerosis cohorts, the prevalence of chronic active lesions at individual patient level and demonstrated that a higher proportion of lesions with innate immune cell activity at the lesion rim was associated with an increased clinical disease severity.

## Materials and methods

### Study subjects

A total of 91 multiple sclerosis patients were imaged. Of them, 67 had RRMS and 24 had SPMS. For comparison, 18 age- and sex-matched healthy control (HC) persons were included. The patients were recruited from the outpatient clinic of the Division of Clinical Neurosciences at the University Hospital of Turku, Finland. The requirements for inclusion were willingness to participate in a PET study and multiple sclerosis diagnosis according to the McDonald criteria 2017.^22^ All participants provided written informed consent and the study was conducted according to the Declaration of Helsinki, with approval by the Ethics Committee of the Hospital District of Southwest Finland.

Clinical relapse and/or corticosteroid treatment within 30 days of evaluation and gadolinium contrast enhancement in conventional MRI (cMRI) were considered as exclusion criteria to avoid confounding effects of acute inflammation on the innate immune cell activation and chronic lesion characterization. Exclusion criteria also included inability to tolerate PET or cMRI, current pregnancy, active neurological or autoimmune disease other than multiple sclerosis or another comorbidity considered significant. The disease severity was evaluated by experienced clinicians using the Expanded Disability Status Scale (EDSS) score and a standardized examination form, the Neurostatus (neurostatus.net).

### MRI acquisition, MRI data analysis and creation of individual lesion core and rim ROIs

**Figure 1:**
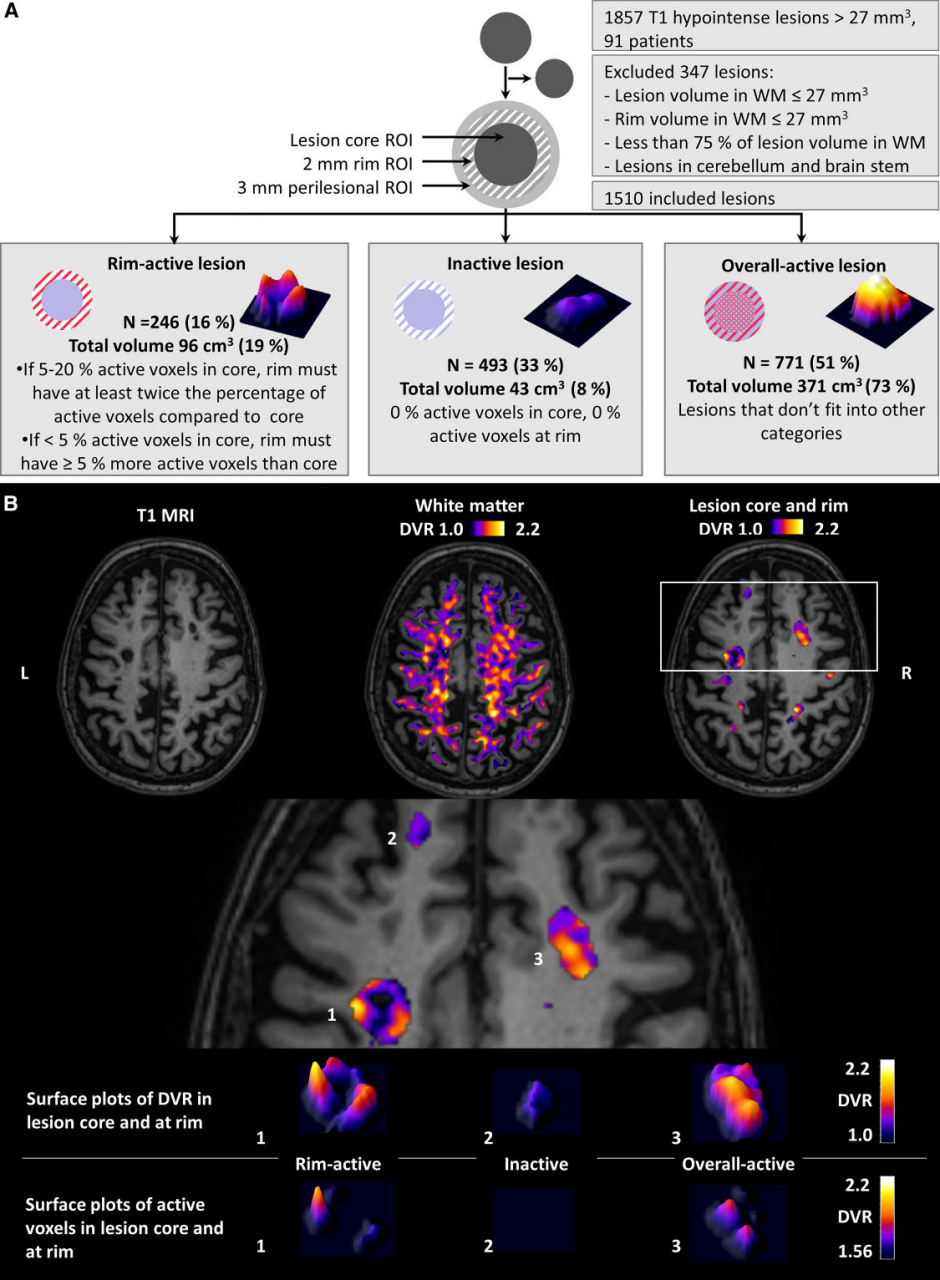
**Flow chart of lesion phenotype analyses and illustrative MRI and PET images**. (**A**) The flow chart illustrates the criteria for lesion inclusion and principles of the lesion classification. Lesion classification is based on proportions of active voxels in the lesion core and at rim. Rim-active lesions contain at least double the proportion of active voxels at the rim compared to core if 5–20% of voxels in the core are active. Rim-active lesions have at least 5% point higher proportion of active voxels at the rim compared to the core, if <5% of the voxels in the core are active. Inactive lesions have 0% of active voxels in the core and at rim. Lesions which do not fit into the other two categories are classified as overall-active lesions. Of all included lesions, 16% were rim-active, 33% were inactive and 51% were overall-active. The DVR distribution of each lesion type is visualized with 3D surface plots. (**B**) T1 MR-image from an SPMS patient (*top left*) with the corresponding parametric PET ^11^C-PK11195 DVR images of the white matter (*top middle*) and the focal T1 lesions (*top right*). The bottom panel highlights the DVR values of selected lesions as 3D surface plots. The bottom row visualizes the voxels defined as active, with DVR > 1.56 (for more details, see the ‘Materials and methods’ section). The colour bar of the PET images shows the dynamic range of DVR in the images.

cMRI with a 3 T Ingenuity TF PET/MR scanner (Philips) was performed for the evaluation of multiple sclerosis pathology and for the acquisition of anatomic reference for the PET images. cMRI sequences were used as previously described.^23^ A semi-automated method was used first to create combined T2 lesion region of interest (ROI) mask image using the Lesion Segmentation Tool (LST, www.statistical-modelling.de/lst.html, a toolbox running in SPM8)^24^ and Carimas (https://turkupetcentre.fi/carimas/) for manual editing as described previously.^19^ A combined T1 lesion ROI mask image was manually shaped slice by slice. The resulting T1 lesion ROI mask image was used to fill the corresponding T1 image with the lesion-filling tool in LST. The filled T1 was then used for segmenting grey matter, white matter and thalamus with the Freesurfer 5.3 software (http://surfer.nmr.mgh.harvard.edu/). The total T1 lesion load was measured from the manually edited T1 ROI masks. The individual T1 lesion core ROI masks were created by separation of individual lesions from the combined T1 lesion masks. Lesions ≤27 mm^3^ in size were excluded to avoid inclusion of unspecific T1 hypointensities. This resulted with a total of 1857 T1 lesions (Fig. 1A).

A 2-mm lesion rim ROI was created by dilating the T1 lesion core ROI by two voxels from the lesion edge and then removing the lesion core ROI (Fig. 1A). This resulted with a lesion rim ROI of width of two voxels extending out from the T1 lesion edge. A 3-mm perilesional ROI was created similarly by dilating the T1 lesion core ROI mask image by three voxels from the T1 lesion edge and then removing the lesion core ROI from the resulting image. In addition, NAWM ROI was created by removing the combined T2 lesion ROI from the white matter ROI. Of the 1857 T1 hypointensities >27 mm^3^ in size, 347 were discarded (those with <75% of the lesion volume in the white matter, those with ≤27 mm^3^ of the rim in the white matter and lesions in the cerebellum and brain stem). This resulted with 1510 lesions for the final evaluation (Fig. 1A). The pre-selection of lesions according to location was done to ensure good-quality PET analysis, given that the high-resolution research tomograph (HRRT) PET scanner resolution is 2.5 mm, and to avoid any potential disturbance related to infratentorial artefacts.

### PET image acquisition

The radiochemical synthesis of ^11^C-PK11195 has been previously described by Rissanen *et al*.^19^ The mean injected dose was 476 ± 52 MBq [mean ± standard deviation (SD)] for the multiple sclerosis patient group and 490 ± 16 MBq for the HC group with no significant dose differences between the groups. PET scan was performed using a brain-dedicated ECAT HRRT scanner (CTI/Siemens) with an intrinsic spatial resolution of 2.5 mm. A 60 min dynamic PET scan was started simultaneously with an intravenous bolus injection of the ^11^C-PK11195 radioligand. Prior to the ligand infusion, a 6 min transmission scan for attenuation correction was obtained using a 137Cs point source. Thermoplastic head mask was used to minimize the movement.

### PET image post-processing and analysis

PET images were reconstructed using 17 time frames as described previously.^19^ The reconstructed PET images were smoothed using a Gaussian 2.5 -mm post-reconstruction filter.^16,19^ Possible displacements between frames were corrected using mutual information realignment in SPM8. Finally, PET images were coregistered to T1 MRI and resampled to match the MRI voxel size 1 mm × 1 mm×1 mm. Innate immune cell activation was evaluated as specific binding of ^11^C-PK11195 using distribution volume ratio (DVR) in pre-specified ROIs. For the estimation of the ^11^C-PK11195 DVR, the time–activity curve corresponding to a reference region devoid of specific TSPO-binding was acquired for each PET session using a supervised cluster algorithm with four predefined kinetic tissue classes (SuperPK software).^25,26^ The reference tissue–input Logan method with a time interval from 20 to 60 min was applied to the regional time–activity curves using the supervised cluster algorithm grey reference input. For the individual lesion DVR analysis, the voxel-wise parametric binding potential (BP_ND_) maps were calculated using basis function implementation of SRTM14 with 250 basis functions. Lower and upper bounds for theta were set to 0.06 and 0.8 1/min. The resulting parametric maps were normalized to Montreal Neurological Institute (MNI) space (MNI database) in SPM8 and the BP_ND_ images were transformed to DVR (DVR = BP_ND_ + 1).

### Individual lesion evaluation for innate immune cell activation

The mean DVR ± SD of all white matter voxels was calculated from all individual DVR images of HC subjects (HC; *n* = 18). Thereafter, 95% confidence interval threshold (HC mean + 1.96 × SD) was used to describe anomalously high voxel activity in lesion phenotyping. Consequently, for each subject, binary TSPO-active voxels were characterized from the DVR images exceeding this threshold (DVR value of 1.56 in our data). Clusters below three connected voxels were excluded in the TSPO-activity image for preventing the inclusion of random peak values.

The proportion of the active voxels in the lesion core ROI and at the rim ROI was used to classify lesions into the following three subtypes: (i) rim-active lesion: lesions with <5% active voxels in the core and at least 5% point higher proportion of active voxels at the rim compared to the core and lesions which have 5–20% active voxels in the core and at the same time at least double the proportion of active voxels at the rim; (ii) inactive lesions: lesions with no active voxels at the rim or in the core and (iii) overall-active lesions: lesions which do not fit into the other two categories (Fig. 1A and B).

### Statistical analysis

The statistical analyses were performed using R (version 4.0.3). Variables are reported as median [interquartile range (IQR)] unless otherwise stated. The Wilcoxon rank-sum test was used to assess the differences in DVRs, counts, proportions, volumes and volume proportions between the two different groups. Spearman’s correlation coefficients were calculated to evaluate the relationships between the continuous variables. Fisher’s exact test was used to compare the gender distributions between two groups and to compare distributions of plaque types in different group variables. The EDSS score was used to classify patients into two groups: <4 and ≥4. The relationships between the volume of the rim-active lesions and EDSS and brain volume were modelled using the multiple linear regression with EDSS and brain volume as outcome variables. The multiple linear regression model was used to assess contribution of rim-active lesion load to brain volume and EDSS. Gender, age, therapy, disease duration and rim-active lesion volume were used as predictive variables in all regression models. Disease-modifying therapies at the time of PET scanning or at most 2 months before were categorized into two classes.^27^ Rim-active lesion volume was included as its logarithm to make the models valid considering the assumptions of the multiple linear regression. All tests were two-tailed and a *P*-value of <0.05 was considered statistically significant for all analyses.

### Data availability

Anonymized data not published within the article will be shared over the next 3 years upon request from a qualified investigator.

## Results

The clinical demographic and radiographic data are given in Table 1. Of the 91 patients included, 67 (74%) had RRMS and they were younger, had shorter disease duration and lower EDSS score and Multiple Sclerosis Severity Score (MSSS) compared to those with secondary progressive disease (*n* = 24, 26%). The mean age of all patients was 44.9 ± 9.7 years (mean ± SD) and their disease duration was 12.5 (±7.7) years. Their median EDSS score was 3.0 (IQR: 2.0–3.5) and median MSSS was 3.9 (IQR: 2.39–5.20). In addition to disease type comparison, patients were divided into two groups based on their EDSS score. Those with EDSS ≥ 4 (*n* = 22, 24%) were older and had longer disease duration compared with those with EDSS < 4 (*n* = 69, 76%).

**Table 1:** Demographic information and imaging parameters

		HC	MS	HC versus MS	RRMS	SPMS	RRMS versus SPMS	EDSS < 4	EDSS ≥ 4	EDSS < 4 versus ≥ 4
Subjects	*N*	18	91		67	24		69	22	
Female	*N*_F_	13	70	0.8	54	16	0.17	53	17	1
	%	72	77		81	67		77	77	
Age (years)	Mean	42.9	44.9	0.5	42.6	51.2	<0.001	43.2	50.2	0.007
	SD	11.4	9.67		8.73	9.53		9.18	9.38	
Years since multiple sclerosis onset	Median		12.1		10.0	17.7	<0.001	10.3	16.0	0.001
	IQR		7.25–15.8		6.55–13.8	12.5–22.9		6.64–13.8	12.3–22.7	
Disease-modifying therapy^a^										
No therapy	*n* (%)		43 (47)		24 (36)	19 (79)	<0.001	27 (39)	16 (73)	0.007
Moderate efficacy therapy	*n* (%)		48 (53)		43 (64)	5 (21)		42 (61)	6 (27)	
NAWM volume (cm^3^)	Mean	492	457	0.014	466	433	0.074	470	416	0.002
	SD	46.9	64.3		62.8	63.4		59.5	62.2	
NAWM volume (PF)	Mean	0.35	0.33	0.007	0.33	0.32	0.075	0.34	0.31	0.016
	SD	0.027	0.034		0.027	0.050		0.027	0.049	
Cortical GM volume (cm^3^)	Mean	464	434	0.048	443	408	0.004	444	402	0.001
	SD	60.8	46.8		42.0	50.9		40.8	51.5	
Cortical GM volume (PF)	Mean	0.33	0.31	0.002	0.32	0.30	0.025	0.32	0.30	0.037
	SD	0.022	0.025		0.020	0.033		0.020	0.033	
T1 lesions >27 mm^3^	N_T1_		1857		1215	642		1208	649	
Per patient	*N*_T1_/*n*		20.4		18.1	26.8		17.5	29.5	
T1 lesion volume (cm^3^)	Median		2.81		2.35	8.18	<0.001	2.35	9.18	<0.001
	IQR		1.48–7.60		1.17–4.30	4.35–17.5		1.20–4.36	4.53–18.2	
EDSS	Median		3.0		2.5	6.0	<0.001	2.5	6.0	<0.001
	IQR		2.0–3.5		2.0–3.0	3.9–6.5		2.0–3.0	5.1–6.5	
MSSS	Median		3.90		3.45	6.24	<0.001	3.17	6.85	<0.001
	IQR		2.39–5.20		2.24–4.74	3.81–8.13		2.15–4.74	4.35–8.18	

The gender and therapy comparison of *P*-values are from Fisher’s exact test. All other tests are the Wilcoxon rank-sum test due to non-normality of the data. EDSS, Expanded Disability Status Scale; GM, grey matter; HC, healthy control; IQR, interquartile range; MSSS, Multiple Sclerosis Severity Score; NAWM, normal appearing white matter; RRMS, relapsing–remitting multiple sclerosis; SD, standard deviation; SPMS, secondary progressive multiple sclerosis.

a Within 2 months prior to the PET scanning.

### Brain ^11^C-PK11195-binding in multiple sclerosis patients and in HCs

**Figure 2:**
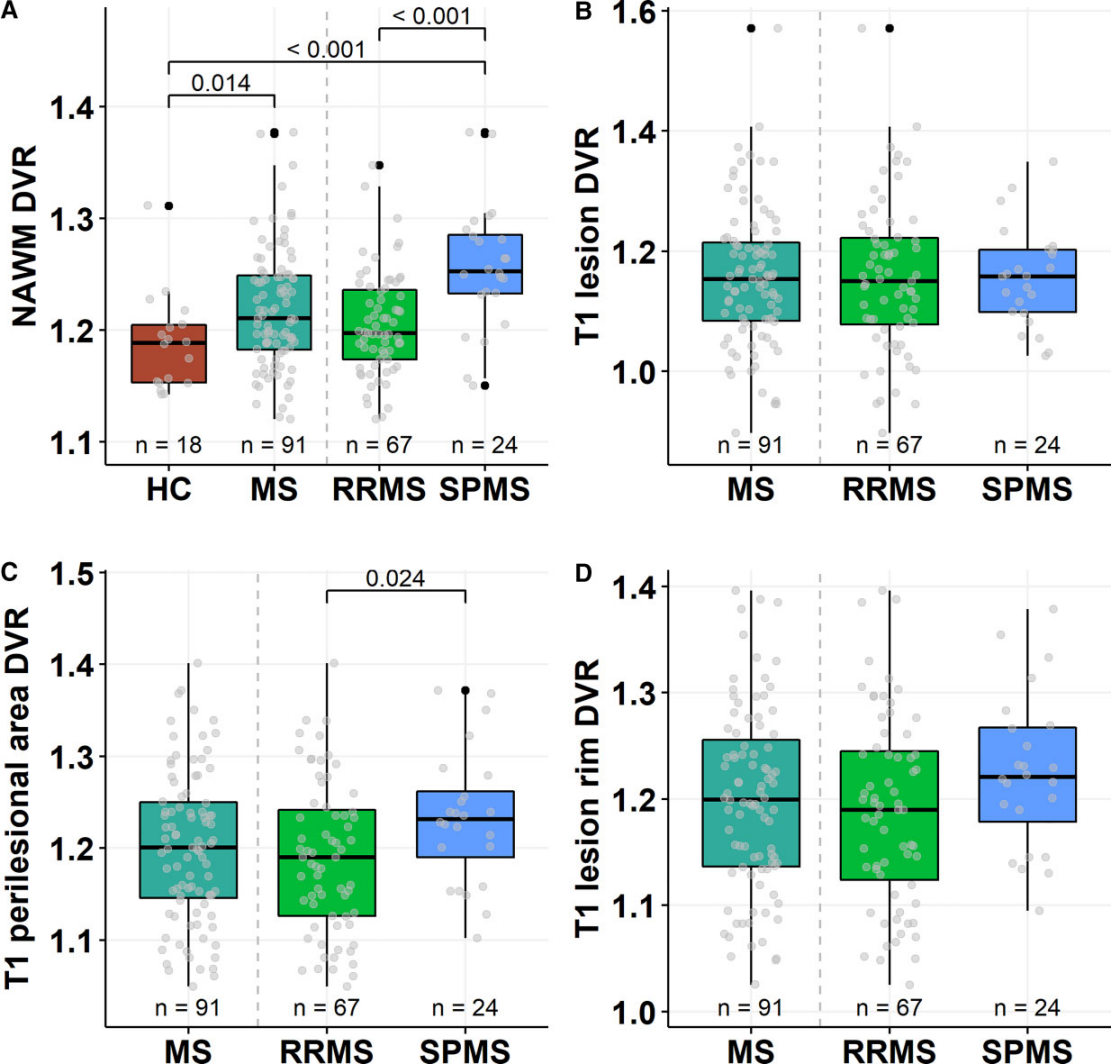
**Brain innate immune cell activation in multiple sclerosis patients and healthy controls**. Box plots of the ^11^C-PK11195 DVR values representing the innate immune cell activation in the white matter of healthy controls and in the NAWM, and in association with lesions in the multiple sclerosis cohort. The Wilcoxon rank-sum test was used for statistical analyses. In box plots, the thick horizontal lines represent the medians, the boxes represent the IQR and the end of the whiskers or the points of the outliers represent the minimum and maximum values.

^11^C-PK11195 radioligand binding in the white matter of HCs was significantly lower (1.19 ± 0.04) compared to the NAWM of all multiple sclerosis patients (1.22 ± 0.05; *P* = 0.014) or SPMS patients separately (1.26 ± 0.06; *P* < 0.001). The innate immune cell activation was higher in SPMS patients compared with RRMS patients both in the NAWM (1.26 ± 0.06 versus 1.21 ± 0.05; *P* < 0.001) and in the 0–3 mm perilesional area surrounding lesions (1.23 ± 0.07 versus 1.19 ± 0.08; *P* = 0.024). The DVR values within the combined T1 lesion ROI or at the combined T1 rim ROI were similar among the disease subtypes (Fig. 2).

### Association of brain volumetric parameters and innate immune cell activation with clinical disability

**Figure 3:**
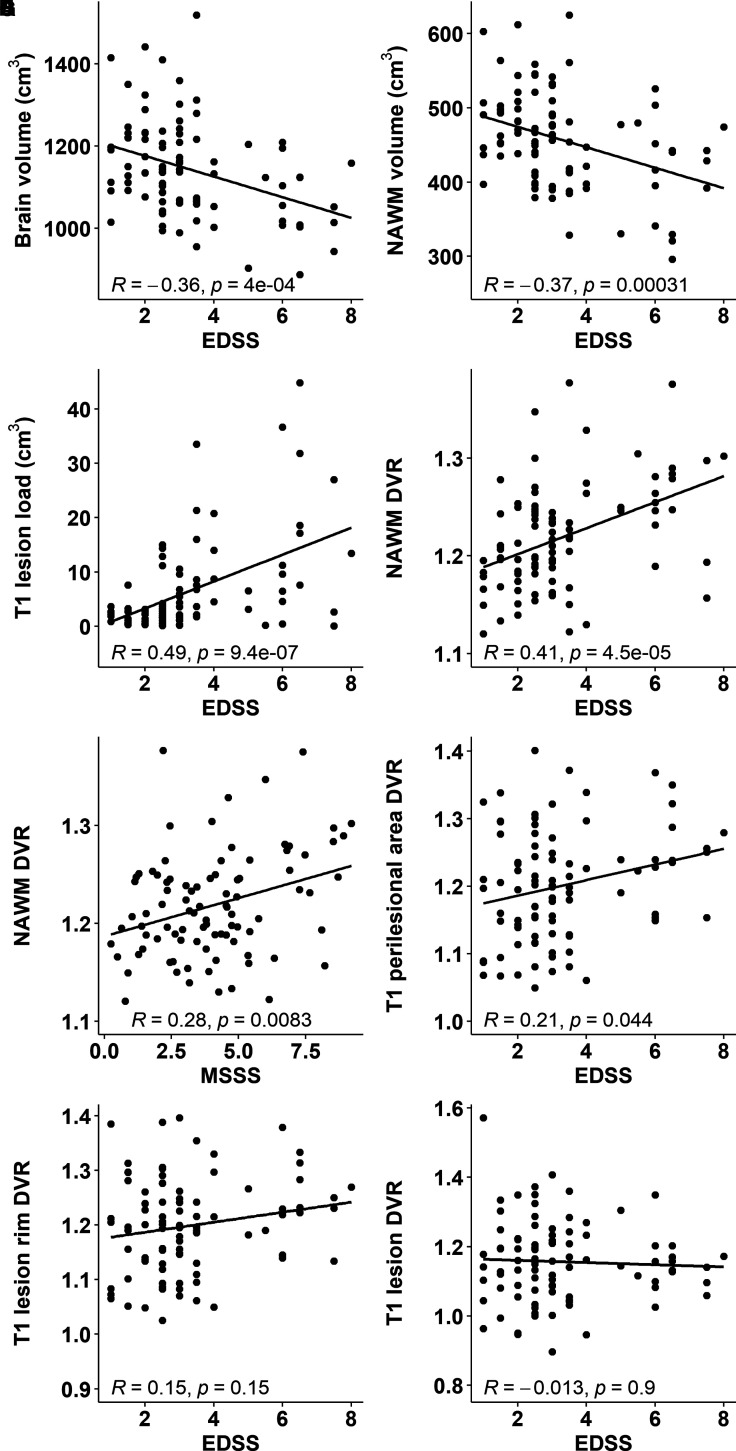
**Association of volumetric parameters and innate immune cell activation with clinical disability**. Smaller brain (**A**) and NAWM volume (**B**) and larger T1 lesion load (**C**) associate with worse clinical disability measured with the EDSS. Higher ^11^C-PK11195 DVR in the NAWM associates with worse disability (**D**) and disease severity (**E**). In addition, higher radioligand binding in the perilesional area correlates with worse disability (**F**). Innate immune cell activation at lesion rim (**G**) or within T1 lesions (**H**) do not associate with the EDSS. Here, ROIs encompassing the entire combined lesion volume or combined perilesional volume were evaluated.

Smaller whole brain and NAWM volume and higher T1 lesion load were significantly associated with higher clinical disability measured using EDSS (Fig. 3A–C). Higher DVR in the NAWM associated with both higher EDSS score (*R* = 0.41, *P* < 0.001) and MSSS (*R* = 0.28, *P* = 0.0083; Fig. 3D and E). Higher DVR values in the 0–3 mm perilesional area correlated with EDSS values (*R* = 0.21, *P* = 0.044; Fig. 3F) but no correlations were observed between the combined T1 rim ROI or the combined T1 lesion ROI DVRs and EDSS values (Fig. 3G and H).

### Distribution of lesions according to innate immune cell activation at rim

Of the 1510 lesions in the final analysis, 246 (16%) were rim-active lesions with a total lesion load of 96 cm^3^ and 493 (33%) were inactive lesions, 43 cm^3^. Overall-active lesions (*n* = 771, 51%) that do not fit into the other two categories had the largest total lesion load of 371 cm^3^ (Fig. 1A). The average (±SD) number of rim-active lesions per patient was 2.7 ± 3.3 (median 2, IQR: 0–4, range: 0–18). A total of 28 patients (31%) did not have rim-active lesions. The average number of inactive lesions was 5.4 ± 4.0 (median 5, IQR: 2.5–7.5, range: 0–22). Six patients (7%) had no inactive lesions (Table 2). Nearly all patients (96%) had overall-active lesions (Table 2). The average number of overall-active lesions per patients was 8.5 ± 7.7 (median 6, IQR: 3–11.5, range: 0–34).

**Table 2:** T1 lesions according to innate immune cell activation in various clinical multiple sclerosis subgroups

		All	RRMS	SPMS	RRMS versus SPMS	EDSS < 4	EDSS ≥ 4	EDSS < 4 versus ≥ 4
Number of patients, *n*		91	67	24		69	22	
	Patients with rim-active lesions, *n* (%)	63 (69)	41 (61)	22 (92)		43 (62)	10 (91)	
	Patients with inactive lesions, *n* (%)	85 (93)	65 (97)	20 (83)		67 (97)	18 (82)	
	Patients with overall-active lesions, *n* (%)	87 (96)	64 (96)	23 (96)		66 (96)	21 (95)	
Average number of lesion subtypes per patient (median, IQR)	Total	12 (7–22)	11 (7–20.5)	20 (10.5–26)	0.063	12 (7–20)	20.5 (9–34.25)	0.073
	Rim-active	2 (0–4)	1 (0–3)	3 (1–4)	0.029	1 (0–3)	3 (1.25–6.5)	0.005
	Inactive	5 (2.5–7.5)	5 (3–7)	4 (1.75–9.25)	0.8	5 (3–7)	4 (1.25–7.75)	0.5
	Overall-active	6 (3–11.5)	6 (2–11)	10.5 (4.75–14.5)	0.051	6 (2–11)	11 (3.25–20.5)	0.033
Proportions of lesion subtypes per patient (%)	Rim-active	13 (0–21)	10 (0–20)	19 (11–26)	0.009	10 (0–20)	23 (12–28)	0.001
	Inactive	38 (21–56)	40 (23–61)	27 (12–46)	0.029	40 (24–60)	23 (12–41)	0.004
	Overall-active	48 (35–60)	47 (33–60)	51 (39–67)	0.3	45 (33–60)	52 (43–65)	0.11
Average lesion subtype volumes per patient (median, IQR)	Total	2.21 (0.98–6.91)	1.77 (0.77–3.29)	7.20 (4.04–16.0)	<0.001	1.77 (0.80–3.43)	7.92 (3.94–17.5)	<0.001
	Rim-active	0.13 (0–0.40)	0.06 (0–0.25)	0.41 (0.15–3.18)	<0.001	0.06 (0–0.26)	0.47 (0.25–4.66)	<0.001
	Inactive	0.40 (0.14–0.61)	0.41 (0.17–0.60)	0.26 (0.11–0.63)	0.5	0.40 (0.18–0.59)	0.25 (0.10–0.67)	0.5
	Overall-active	1.42 (0.29–5.81)	1.18 (0.22–2.44)	5.95 (2.51–9.76)	<0.001	1.23 (0.22–2.89)	6.32 (1.93–8.79)	0.002
Proportions of lesion subtype volumes per patient (%)	Rim-active	4.3 (0–16)	2.4 (0–10)	13 (3.2–34)	0.001	2.4 (0–10)	13 (5.4–39)	<0.001
	Inactive	18 (4.4–38)	23 (6.8–56)	4.9 (1.0–22)	0.002	23 (6.8–51)	4.3 (1.0–21)	0.003
	Overall-active	67 (43–83)	68 (40–82)	65 (50–86)	0.5	69 (41–84)	64 (46–81)	0.8
Average number of active voxels at rim^a^	Rim-active	92 (47–203)	74 (43–162)	158 (76–944)	0.022	73 (42–144)	210 (90–1243)	0.003

The Wilcoxon rank-sum test has been used to compare the groups due to non-normality of the data. All significant differences in comparisons remained significant after multiple comparison corrections, using the false discovery rate method for the number of different tests in the groups being compared (*n* = 15). Lesion volumes in millilitres (cm^3^), unless otherwise stated. Only white matter parts of the lesions with >75% of the volume in white matter are included. Only lesions with core and rim volumes >27 cm^3^ in white matter are included. Values are expressed as median (IQR), except for total numbers. EDSS, Expanded Disability Status Scale; IQR, interquartile range; RRMS, relapsing–remitting multiple sclerosis; SPMS, secondary progressive multiple sclerosis.

^a^ Only patients with rim-active lesions included.

### Distribution of lesion phenotypes according to clinical patient profile

Among SPMS patients, the number of rim-active lesions (median 3, IQR: 1–4, range: 0–11) was higher compared to RRMS patients (median 1, IQR: 0–3, range: 0–18, *P* = 0.029; Table 2). In an individual SPMS patient, 19% of T1 lesions were rim-active, 27% were inactive and 51% were overall-active. In RRMS, 10% of lesions were rim-active, 40% were inactive and 47% were overall-active. The fractions of rim-active and inactive lesions between SPMS and RRMS were statistically significantly different (*P* = 0.009 and 0.029, respectively; Table 2). The total T1 lesion load per patient was significantly higher in SPMS [7.20 cm^3^ (4.04–16.0)] compared to RRMS [1.77 cm^3^ (0.77–3.29); *P* < 0.001]. Similarly, the average volume of rim-active lesions per patient was larger among SPMS patients [0.41 cm^3^ (0.15–3.18)] compared to relapsing–remitting [0.06 cm^3^ (0–0.25); *P* < 0.001]. Of the total lesion volume among SPMS patients, 13% belonged to rim-active lesion fraction and 4.9% belonged to inactive lesion fraction, whereas in relapsing–remitting patients, the corresponding proportions were 2.4 and 23%, respectively (*P* = 0.001 and 0.002; Table 2). There were no differences in the volume percentages of overall-active lesions between the multiple sclerosis subgroups. Similar results were obtained when the patients were subdivided based on EDSS scores (≥4 or <4, Table 2).

**Figure 4:**
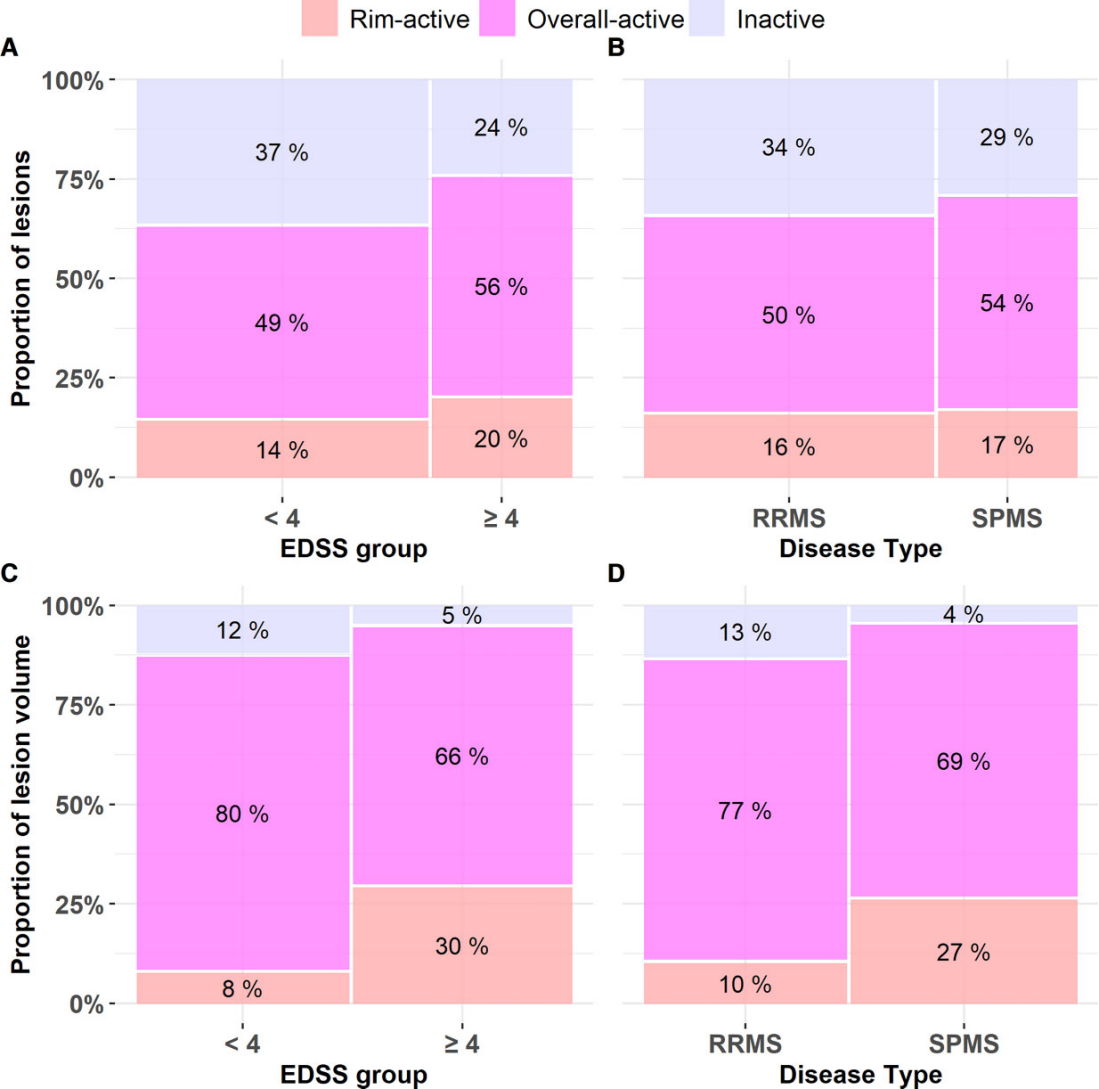
**Fractions of lesion types among multiple sclerosis subgroups**. Proportions of the lesion subtypes differ between patients with EDSS scores of <4 and ≥4 (Fisher’s exact test *P* < 0.001) (**A**) but not between RRMS and SPMS groups (**B**). Proportions of the lesion subtype volumes are different between patients with EDSS scores of <4 and ≥4 (Fisher’s exact test *P* < 0.001) (**C**), and in RRMS versus SPMS (*P* < 0.001) (**D**). The width of the bar represents the number of lesions (**A** and **B**) or lesion volumes (**C** and **D**) within the patient subgroup, and the height of the bar represents the percentage of the lesion type in question (**A** and **B**) or the volume percentage of the lesion type (**C** and **D**), with the exact percentage marked in the respective box. The total lesion numbers in the respective groups were 1020 among patients with an EDSS score of <4 (69 patients) and 490 among patients with an EDSS score of ≥4 (22 patients), 1020 among RRMS (67 patients) and 490 among SPMS (24 patients). The total lesion volumes in the respective groups were 251 cm^3^ among patients with an EDSS score of <4 and 259 cm^3^ among patients with an EDSS score of ≥4, 240 cm^3^ among RRMS and 269 cm^3^ among SPMS.

The frequencies of the different plaque types were statistically and significantly different between patients with EDSS scores of ≥4 and <4 when evaluated using Fisher’s exact test (*P* < 0.001; Fig. 4A). In the patient cohort with an EDSS score of ≥4, the fraction of rim-active lesions was higher (56%) and fraction of inactive lesions was lower (24%) compared to patients with an EDSS score of <4 (49 and 37%, respectively). Similar trend was observed between SPMS and RRMS, but statistical significance was not reached (Fig. 4B). Distributions of volume percentages across the lesion subtypes were different both in EDSS ≥ 4 versus EDSS < 4 and SPMS versus RRMS (*P* < 0.001, Fig. 4C and D).

### Correlation of rim-active lesion load with clinical disability and brain atrophy

**Figure 5:**
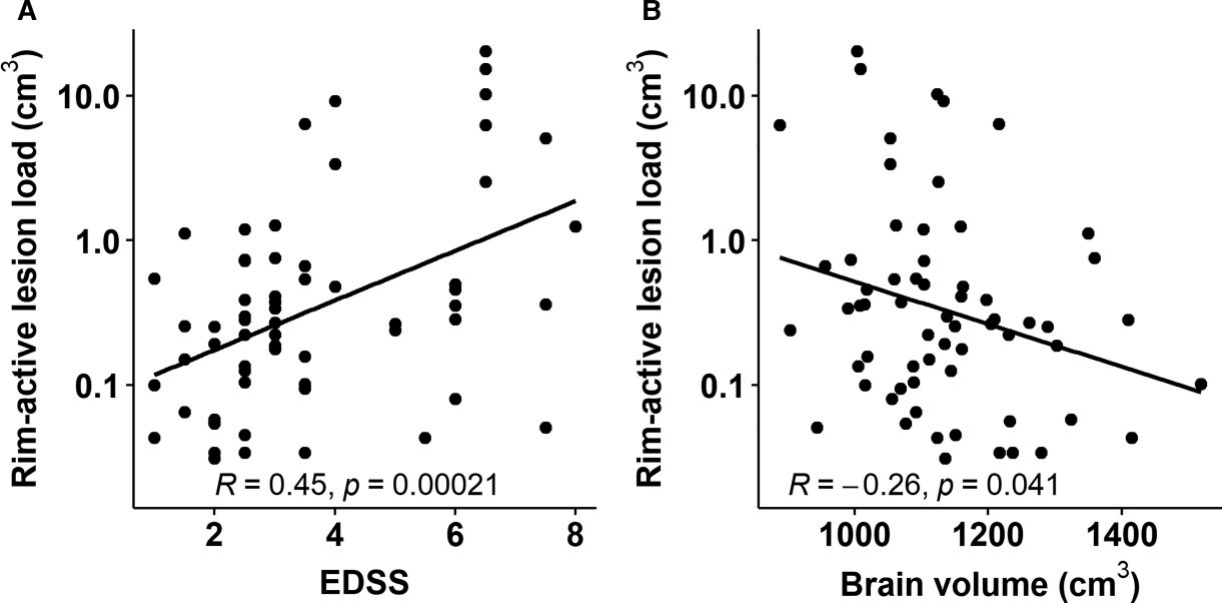
**Rim-active lesion load correlates with clinical disability and brain volume**. The volume of rim-active lesions associates with higher clinical disability (**A**) and greater brain atrophy (**B**). The associations remain significant after multiple linear regression model.

Higher rim-active lesion volume was correlated with higher EDSS (*R* = 0.45, *P* < 0.001; Fig. 5A) and with lower brain volume (*R* = −0.26, *P* = 0.041; Fig. 5B) among the 63 individuals who had rim-active lesions. In the entire cohort, higher number of rim-active lesions correlated with higher EDSS (*R* = 0.31, *P* = 0.003, data not shown). The rim-active lesion associations with higher clinical disability (*P* < 0.001) and brain atrophy (*P* = 0.043) remain significant when taking into account background variables such as gender, age, therapy and disease duration in multiple linear regression.

### Innate immune cell activation at rim is variable but more prevalent among patients with advanced disease

**Figure 6:**
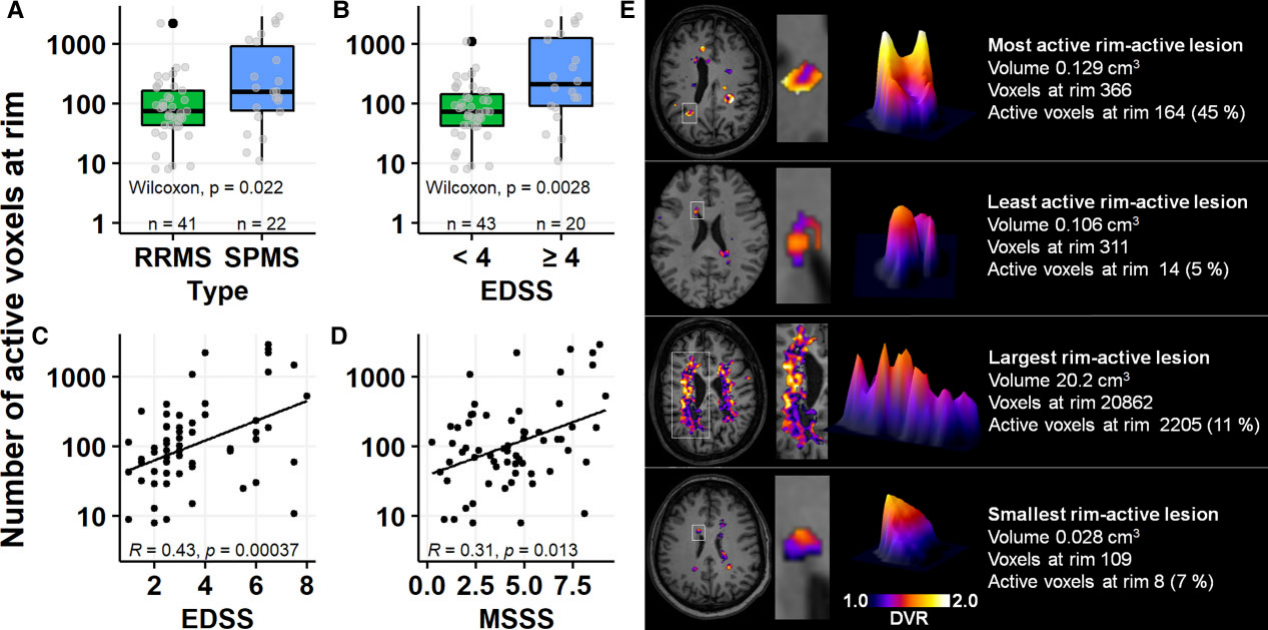
**Innate immune cell activation at rim is variable but more prevalent among patients with advanced disease**. The number of active voxels at the rim was higher among secondary progressive compared to relapsing–remitting patients (**A**) and in patients with an EDSS score of ≥ 4 compared to those with a lower EDSS score (**B**). The number of active voxels at rim correlated with an EDSS-measured disability (**C**) and with an MSSS-assessed disease severity (**D**). (**E)** The great variability in both size and the degree of TSPO-binding of rim-active lesions.

The average active voxel number at the rim-active lesions per patient was 92 (median; IQR: 47–203), and on average, there were significantly more active voxels at the rim in SPMS patients compared to RRMS (median values: 158 versus 74, *P* = 0.022; Fig. 6A and Table 2). Similarly, there was a significantly higher number of active voxels at the rim in patients with EDSS ≥ 4 versus EDSS < 4 (median values: 210 versus 73, *P*  *=* 0.003; Fig. 6B and Table 2). The number of active voxels at rim correlated significantly both with EDSS score (*R* = 0.43, *P* < 0.001) and MSSS (*R* = 0.31, *P* = 0.013; Fig. 6C and D).

The lesion sizes varied greatly within this heterogeneous cohort of multiple sclerosis patients. The smallest lesion (inactive, 0.028 cm^3^ in volume) had 69 voxels at the rim and the largest lesion (20.2 cm^3^ in volume) had 20 862 voxels at the rim. This largest lesion was rim-active with 2205 (11%) active voxels at the rim. The smallest rim-active lesion (0.028 cm^3^) had 109 voxels at the rim, with eight active voxels (7%; Fig. 6E). The proportion of active voxels at the rim of rim-active lesions varied greatly, between 5 and 45% (Fig. 6E).

## Discussion

The results from this comprehensive, cross-sectional study focusing on 91 PET-imaged multiple sclerosis patients and 1510 lesions demonstrate that *in vivo* TSPO-PET can be used to quantify innate immune cell activation at chronic lesion edge with subsequent categorization of the lesions into rim-active and rim-inactive lesions. At patient level, the rim-active lesion fraction was larger among SPMS (19%) compared to RRMS (10%) and according to multiple linear regression modelling, the rim-active lesion load contributed more significantly to clinical disability (*P* = 0.014) than the NAWM DVR (*P* = 0.6; data not shown). The detrimental nature of the rim-active lesions was demonstrated by correlation of rim-active lesion load at rim to brain atrophy measures in MRI. Similarly, patients with an increased disability, older age, and longer disease duration had proportionately more rim-active than rim-inactive lesions and a significant difference was observed in the perilesional microglial activation between RRMS and SPMS patients.^19^ Moreover, higher TSPO-binding in the perilesional NAWM predicted progression during a 4-year follow-up.^28^ In CNS disease, the innate immune system may respond to neuronal injury by activation.^29^ On the other hand, the innate immune system may get arrested in a proinflammatory, neuronal damage promoting phenotype once activated in the context of neuroinflammatory disease. In multiple sclerosis, this may promote a state of self-propagating damage contributing to disease progression and disability accrual.^29^ In line with this, microglial activation in the NAWM was recently shown to co-localize with markers of microstructural damage in an *in vivo* study combining TSPO-PET and DTI-MRI imaging.^23^ In addition, microglial activation has been shown to associate with age and with multiple sclerosis disease duration.^19,30,31^ Due to this phenomenon, we took age and disease duration into account in the model where the rim-active lesion load association with clinical disability and brain atrophy was addressed (Fig. 5).

In the seminal neuropathology work by Frischer *et al.*,^4^ of 2476 white matter plaques, 35% were classified as active plaques and were majorly found only in RRMS patients. A total of 15% of the lesions were smouldering and almost exclusively found in progressive multiple sclerosis. Of the lesions, 35% were inactive and 15% were classified as shadow plaques. In the present work, the lesion distribution was very similar with 16% rim-active (corresponding to smouldering), 33% inactive lesions and 51% overall-active (likely partly corresponding to the pathological classification of active plaques and shadow plaques). In both studies, the average disease duration was 12 years. In the Frischer analysis, half of the lesions were infratentorial, and the plaque type distribution was found to be rather similar between supratentorial and infratentorial lesions. In the present work, all evaluated lesions were supratentorial white matter lesions.

TSPO-PET detects both activated microglial cells and macrophages. In addition, a small proportion (25%) of astrocytes bind the TSPO-ligands.^32^ It is thus impossible to determine for certain the exact cellular correlates of the increased TSPO binding, but a most likely interpretation of our results is that the ligand binding at chronic lesion edge and in the NAWM reflects proinflammatory microglia and macrophage activation with some binding to astrocytes.^33,34^ The lesions with an increased TSPO-binding both in the core and at rim have possibly evolved more recently.^6,35^ The use of novel PET-ligands may assist in more accurate *in vivo* segregation of astrocytes from microglial cells, or M1-type innate immune cells from M2-type innate immune cells, and thus may further improve the specificity of the *in vivo* plaque differentiation in the future.^36,37^

MRI-based techniques which rely on detection of iron within activated microglia and macrophages have been developed to identify chronic active or smouldering lesions *in vivo*.^8,9,11,38–43^ Here, MRI sequences sensitive to tissue susceptibility due to paramagnetic properties of the cells such as high-resolution T2*, susceptibility-weighted imaging, phase MRI using 7 or 3T and quantitative susceptibility mapping have been used.^8,9,11,13,38–44^ Iron rim lesions were detected in 46–81% of the studied multiple sclerosis patients depending on the cohort/study.^10,41,45–48^ Unlike the neuropathological studies,^4,49,50^ some^11,13,45^ but not all^51^ MRI studies of iron rim lesions have detected them more often in RRMS patients compared to progressive multiple sclerosis. Larger MRI studies with more homogeneous methodologies regarding iron rim detection will likely help settle this discrepancy. The smouldering lesions are potentially the ones to expand.^10,45,52^ Our data are in line with this, as in our cohort, the largest lesions were rim-active, and were often located in the periventricular area, and had likely been formed by fusion of several rim-active lesions together, with the largest such confluent lesion having a volume of 20.2 cm3. On the contrary, the largest inactive lesion was only 1.3 cm^3^.

The analysis of hot microglia at rim using TSPO-PET-imaging relies on fully automated, quantitative methodology and allows sensitive detection of the detrimental cell clusters in a three-dimensional space. In this study, a novel approach was used to define the rim as two voxels extending from the T1 lesion edge with the idea to address as limited rim area as possible with taking the resolution of PET scanner into account. In our previous work, the DVR in the 0–6 mm perilesional ROI was different in SPMS versus RRMS.^19^ In the present work, 0–3 mm perilesional ROI was similarly different in RRMS versus SPMS, but in the 2-mm perilesional ROI no difference was observed (Fig. 2C and D). We interpret that the latter is perhaps due to the close vicinity of the lesion core with lower DVR and no differences between RRMS and SPMS. All hemispheric white matter T1 lesions with minimum rim and core size of >27 mm^3^ were included unless they extended to the grey matter by >25%. In some of the MRI studies, only well-demarcated independent lesions have been included in the analysis,^45^ which might have promoted the predominance of iron rim lesions in earlier disease stages. Despite the differences in the methodologies between the MRI and PET, the frequency of the rim-active lesions in the present study, with 16% of all lesions being rim-active in the entire multiple sclerosis cohort, was in a similar range compared to many of the MRI studies.

Taken together, there are now neuropathology-based, susceptibility MRI-based and TSPO-PET-based methods to quantify progression-associated smouldering inflammation in multiple sclerosis brain. Future studies will demonstrate how the different methods can be used in a complementary way to evaluate the dynamic innate inflammatory process contributing to neuroaxonal damage and disease progression. Accurate and dynamic *in vivo* assessment of progression-related innate immune system activation has potential to advance our understanding of the mechanisms related to disability accrual among multiple sclerosis patients. This has implications for predicting future disease course,^28^ for obtaining meaningful outcome markers and for selecting optimal patients when performing treatment trials of progressive multiple sclerosis.

## Abbreviations

BP_ND_ =: binding potential
cMRI =: conventional MRI
DVR =: distribution volume ratio
EDSS =: expanded disability status scale
HC =: healthy control
HRRT =: high-resolution research tomograph
IQR =: interquartile range
LST =: lesion segmentation tool
MSSS =: multiple sclerosis severity score
NAWM =: normal appearing white matter
ROI =: region of interest
RRMS =: relapsing–remitting multiple sclerosis
SD =: standard deviation
SPMS =: secondary progressive multiple sclerosis
TSPO =: 18 kDa translocator protein
